# Paradoxical morphea-like reaction after initiation of dupilumab for nodular prurigo^؆^

**DOI:** 10.1016/j.abd.2025.501193

**Published:** 2025-08-15

**Authors:** José González Fernández, Mariano Ara Martín, Sergio García González, Sara Pilar Martínez Cisneros, Mar García García, Sonia de la Fuente Meira

**Affiliations:** a Department of Dermatology, Lozano Blesa University Clinical Hospital, Zaragoza, Spain; b Instituto de Investigación Sanitaria de Aragón, Zaragoza, Spain; c Faculty of Medicine, University of Zaragoza, Zaragoza, Spain; d Department of Pathology, Lozano Blesa University Clinical Hospital, Zaragoza, Spain

Dear Editor,

Localized morphea, or localized scleroderma, is an autoimmune connective tissue disorder characterized by inflammation and sclerosis of the skin and sometimes deeper tissues. The pathophysiology is not fully understood, likely multifactorial, involving interactions among genetic factors, infections, medications, and immune pathway alterations that promote fibrosis.^1^

A 68-year-old male with a history of epithelioid hemangioma in the proximal right ulna presented with pruritic, excoriated erythematous-violaceous papules and plaques (Fig. 1). Histopathology revealed focal epidermal erosion accompanied by marked epidermal hyperplasia, hypergranulosis, and spongiosis, along with a perivascular and interstitial lymphocytic infiltrate in the superficial dermis; findings consistent with nodular prurigo (Fig. 2). Treatment with dupilumab was initiated with an initial dose of 600 mg, followed by 300 mg administered every two weeks. After six months of treatment, painful skin lesions appeared on the right elbow and trunk. Physical examination revealed an indurated, shiny brownish plaque extending from the mid-arm to the distal third of the forearm on the right upper limb, as well as several oval plaques on both flanks of the trunk (Fig. 3). The patient’s blood count was normal, without eosinophilia, and autoimmunity and serology studies showed no abnormalities.

**Fig. 1:**
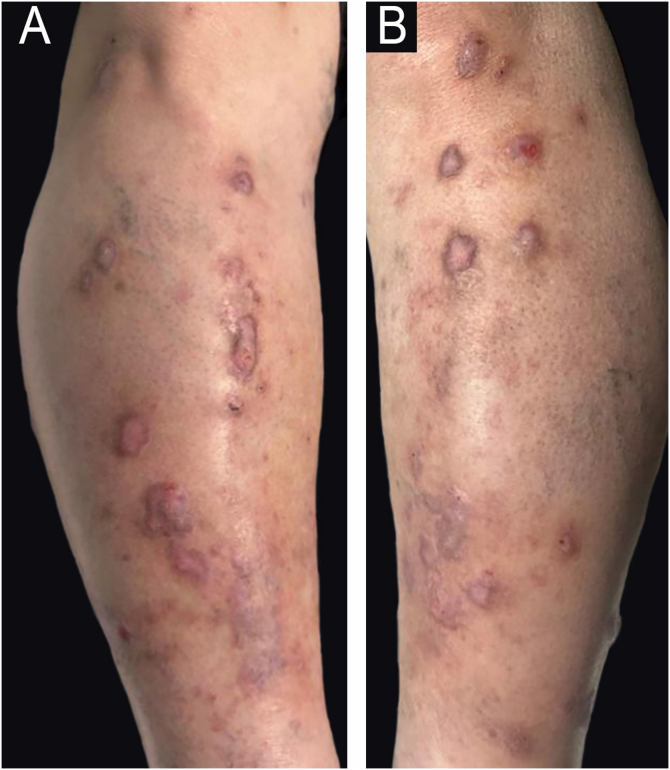
Excoriated bilateral erythematous-violaceous papules and plaques on the pretibial regions.

**Fig. 2:**
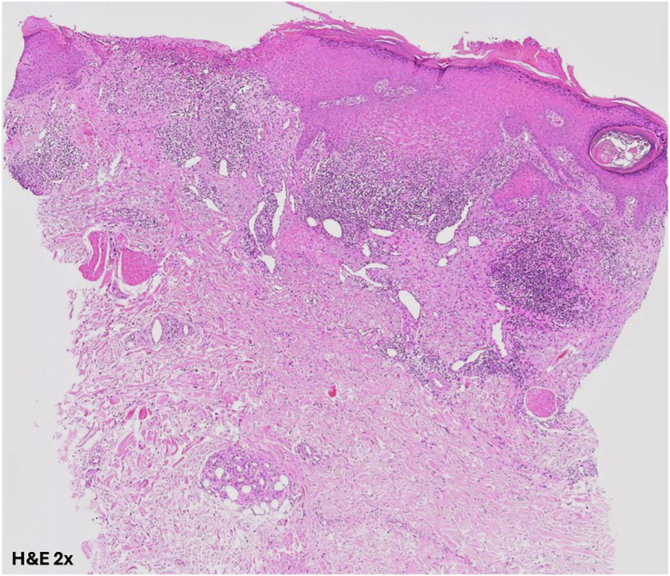
At low power view, histopathology shows an epidermis with acanthosis and focal excoriation (Hematoxylin & eosin, ×20).

**Fig. 3:**
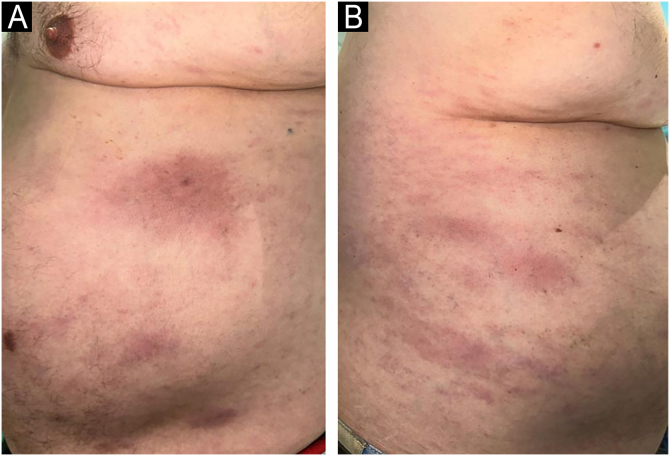
Morphea plaques in the patient. (A and B) Oval erythematous-violaceous plaques on the left and right flanks.

Histopathology of the trunk lesion showed dermal changes, including edema, vascular ectasia, and progressive sclerosis extending into the subcutaneous tissue (Fig. 4). Additionally, a predominantly lymphohistiocytic inflammatory infiltrate was observed, along with perivascular and interstitial involvement. Immunohistochemical staining for CD34 revealed the loss of dermal dendritic cells (Fig. 2).

**Fig. 4:**
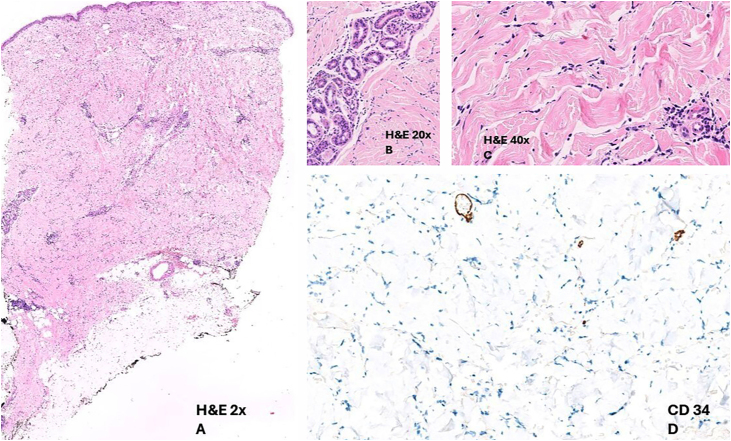
(A) Preserved epidermis and the presence of a perivascular inflammatory infiltrate in the dermis (Hematoxylin & eosin, ×20). (B-C) Eccrine glands without adipose stroma, along with dense collagen. The perivascular infiltrate consists predominantly of lymphohistiocytic elements with occasional plasma cells. This infiltrate is also observed in the interstitium at this magnification (Hematoxylin & eosin, ×200 and ×400). (D) IHC: CD34 staining shows a loss of dermal dendritic cells normally present in healthy skin.

Dupilumab was discontinued, and treatment with weekly methotrexate (15 mg), oral prednisone (tapering regimen), and topical clobetasol (once daily) was initiated, resulting in progressive improvement of the lesions.

Morphea is a multifactorial connective tissue disorder. It is thought to involve two stages of pathophysiology: an early inflammatory stage mediated by the Th1 and Th17 axes, followed by a fibrotic stage mediated by the Th2 axis.^1^ The Th2 response has been implicated in the development of fibrotic disorders by promoting fibroblast proliferation and collagen production, suggesting that blocking the IL-4/IL-13 axis might prevent fibrosis. Dupilumab, a monoclonal antibody targeting the α subunit of the Interleukin-4 Receptor (IL-4Rα), blocks IL-4 and IL-13 signaling and has been proposed as a potential treatment for localized scleroderma.^2^ Although only four cases of dupilumab-associated morphea and localized sclerosis have been reported in the literature to date (Table 1), numerous cases of psoriasiform dermatitis mediated by the Th1 and Th17 pathways have been reported following initiation of dupilumab therapy.^1^, ^3^, ^4^, ^5^ In our case, this may be attributed to the inhibition of IL-4, which can promote overproduction of the IL-4δ2 variant. This IL-4 variant increases levels of IFN-γ and TNF-α, activating the Th1 pathway and inflammation, ultimately facilitating collagen deposition in the extracellular matrix.^3^

**Table 1:** Demographic and clinical characteristics of morphea cases following initiation of dupilumab.

Patient	Age	Sex	Underlying condition	Latency period	Clinical presentation	Treatment
1	10 years	Female	Atopic dermatitis	34 weeks after initiation	A plaque on the ankle and foot	Discontinuation of dupilumab. Initiation of methotrexate and oral prednisone
2	14 years	Male	Atopic dermatitis	5 months after initiation	A linear plaque on the scalp	Discontinuation of dupilumab. Initiation of topical corticosteroids; after failure, methotrexate and intravenous methylprednisolone
3	20 years	Female	Atopic dermatitis	8 months after initiation	Multiple plaques on the upper and lower extremities	Discontinuation of dupilumab. Initiation of mycophenolate mofetil
4	67 years	Female	Asthma and chronic rhinitis	5 days after initiation	Multiple lesions on the trunk	Continued dupilumab. Resolution one week after the second injection

CD34+ dermal dendritic cells are usually distributed throughout the reticular dermis and are thought to play roles in wound healing and maintaining dermal architecture.^6^ The loss of CD34 expression helped distinguish morphea from other dermatoses with lymphohistiocytic infiltrates in the dermis and is a phenomenon seen in disorders involving collagen degeneration, such as morphea.^7^, ^8^ CD34 expression in the dermis is inversely proportional to the extent of morphea; thus, in a case like ours, where CD34 expression was lost, there is a higher likelihood of deep tissue involvement. This overlap between deep morphea and eosinophilic fasciitis has been noted.^6^ Given the absence of dermal eosinophils and a normal blood count, a diagnosis of morphea was established. Methotrexate and corticosteroids were chosen for treatment due to their efficacy in both nodular prurigo and morphea.

In conclusion, we present a case of generalized morphea following the initiation of dupilumab for nodular prurigo. Blocking IL-4 and IL-13 represents a potential target for treating sclerotic disorders; however, this blockade may disrupt healing mechanisms, favoring collagen deposition, and increase scar tissue through dysregulation of Th1/Th2 signaling pathways.

## Research data availability

Does not apply.

## Scientific Associate Editor

Hiram Larangeira de Almeida Jr.

## Financial support

None declared.

## Authors’ contributions

José González Fernández: Manuscript preparation and supervision; final manuscript approval.

Mariano Ara Martín: Intellectual participation in the diagnostic and therapeutic management of the case; manuscript review.

Sergio García González: Manuscript preparation; participation in guiding the case.

Sara Pilar Martínez Cisneros: Concept and planning of the study; literature review.

Mar García García: Literature review; manuscript review.

Sonia de la Fuente Meira: Manuscript preparation; final manuscript approval; final decision on the diagnostic and therapeutic management of the case.

## Conflicts of interest

None declared.
